# Botanical Description of British Plants

**Published:** 1807-01

**Authors:** 


### *Botanical Description of British Plants.*

[ Continued from vol. xvi. page 527—562. ]

5. NYMPHÆA. *N. lutea.*

*Ang.* Yellow water-lilly. Watercan.

*Gen. Desc.* Bloss. many-pet. Cal. four or five leav. Sum. round, flat, sitting. Berry superior, lopped, many-celled.

*Spec. Desc.* *Leaves* heart-shaped, entire, a deep notch at the base. *Cal.* five-leaved, larger than the petals, leaves roundish, yellow, outside at the base green. *Petals* small, fleshy, yellow. *Stam.* after shedding, bent back. *Seed-vessel*

*vessel* often fifteen celled, or more. *Flowers* on long fruit stalks. *Slow rivers, pools, ditches, in the sea at Cork.* Bl. July, Aug.

*Use.* An infusion of one pound of the fresh root to one gallon of water, taken in the dose of a pint night and morning, cured a *leprous* eruption of the arm. Ray observes that the flowers smell like brandy.—*Withering.* The roots rubbed with milk destroy crickets and cockroaches. Swine eat of it; goats are not fond of it; horses, cows, and sheep refuse it.—*Linnæus.*

6. Nymphæa. *N. alba.*

*Ang.* White Water-lilly. Watercan. Candoch. Water-socks.

*Gen. Desc. As above.*

*Spec. Desc. Leaves* oval, heart-shaped, with a deep notch at the base, *v.* entire, *cal.* four-cleft, leaves smaller than the outer petals. *Petals* in several rows, resembling a double flower, bright white; flower opens about 7 A. M. closes about 4 P. M. and then lies down on the water. *Leaf* and *fruit stalks* round, full of pores within. *Summits* seventeen or eighteen, in a circle, corresponding with as many cells in the germen. *Stam.* fixed to the side of the germen. *Slow rivers, ponds. In the sea, at Cork.* Bl. July.

*Use.* The roots are used in Ireland, and in the island of Java, to dye a dark *brown.* This is one of the most beautiful of the English plants; and, for the splendour of its starry blossom, brilliant white, and natural double, well deserves to be cultivated by the curious florist: it may be propagated by transplanting in winter its roots, which are bulbous. It extends itself by long runners, which form a root at the end, and send up leaf-stalks in deep water. The petals gradually lessen as they approach the centre of the flower, where the outer filaments, expanding in breadth, gradually assume the form of petals, as is generally the case in the double flowers of the garden. Swine eat it; goats are not fond of it; horses and cows refuse it.—*Withering.*

7. Tilia. *T. europæa. T. fæmina.*

*Ang.* Lime tree. Linden tree.

*Gen. Desc.* Bloss. five-pet. cal. five div. Seed vess. leather-like, globular, five-celled, five-val. opening at the base.

*Spec. Desc. Flowers* without nectary. *Berry* four-celled. *Leaves* heart-shaped, serrated. *Floral-leaf* yellow-green, nearly as long as fruit-stalk, and attached to it half its length. *Bloss.* whitish. *Woods and hedges.*

*Use.* The sap inspissated, affords a considerable quantity of sugar. The leaves are dried in some countries for the winter food of sheep and goats; they are eaten by cows in the autumn, but they give a bad taste to the milk. The flowers are flagrant, and afford the best honey for bees. The bark, macerated in water, may be manufactured into ropes and fishing-nets. The wood is soft, light, and smooth; close grained, and not subject to the worm: it makes good charcoal for gunpowder and for drawing; it is used for leather-cutter's boards, and for carved work, and is also employed by the turner. This tree flourishes best on the sides of hills, but will grow very well in meadow grounds. It is useful for forming shady walks and clipped hedges; is easily transplanted, and does not destroy the grass beneath it.—*Withering.*

Polyandria. Digynia.

8. Poterium. *P. sanguisorba. Pimpinella minor.*

*Ang.* Upland Burnet.

*Gen. Desc.* Monæceous. Cal. four-leaved; Bloss. four-div. M. stam. thirty to forty. Fem. drupa juiceless, beneath, one or two-celled, formed of the indurated tube of the blossoms.

*Spec. Desc.* Thornless, *Stem* angular. *Barren fl.* has two feeble pistils. *Berry* dry, angular; seeds four-cornered, tapering. *F. fl.* at the top of the spike. *Bloss.* greenish, sometimes purplish on the outside. *In dry calcareous soil.* Bl. April, May.

*Use.* The leaves and seeds are mildly *astringent,* and have been used in *dysenteries* and *hæmorrhages. Lewis.* The young leaves are frequently used in sallads and cool-tankards: when bruised they smell like the cucumber. It has of late years been cultivated by farmers, as affording food for cattle early in the spring; and, from its very luxuriant growth, allowing of three mowings during the summer; but it did not answer the expectations formed of it, and is now generally laid aside. Cattle are said not to be fond of it, and its produce is not sufficient to answer the expences of its culture.—*Curtis.* On Salisbury Plain it forms almost the whole staple of the herbage over a great extent of that most excellent sheep-walk; it is kept close sheered by the large flocks which pasture there, and appears to be a most valuable plant in hard stocked sheep pastures. By cows it is preferred to clover; but not by sheep or horses.—*Mr. Pitt.* As it appears only to love a calcareous soil, it may have failed in cultivation from want of of attention being paid to that circumstance; and, when fully grown, it may be disliked by cattle, though, when close bitten, so valuable to sheep.—*Withering.*

Polyandria. Trigynia.

9. Delphinium. *D. consolida. D. vulgare. Consolida hortensis.*

*Ang.* Wild Larkspur. Lark's-heel. Lark's-claw. Lark's-toes.

*Gen. Desc.* Cal. 0. pet. five or six; nect. cloven, horn shaped behind; caps. leguminous, many-seeded.

*Spec. Desc.* *Capsule* single. *Nectary* one leaf. *Stem.* sub-divided. *Leaves* deeply divided into three or five parts, these deeply cut into slender strap-shaped segments, often forked at the end. *Petals* irregularly scolloped, lateral ones broadest, uppermost spear-shaped, blue; by cultivation white, purple, red, or bay. *Nect.* within upper petal, tube projecting backwards within the tube of that petal. *Anth.* double, yellow. *Germ.* conical, woolly. *Style* 0. *Summ.* two; white, small, fleshy, flatted. *Corn-fields.* Bl. June, Sept.

*Use.* The seeds are acrid and poisonous. The expressed juice of the petals, with the addition of a little allum, makes a good blue ink. By cultivation the flowers often become double. Sheep and goats eat it; horses are not fond of it; cows and swine refuse it.—*Withering.* From a foreign species of this plant is produced the stavesacre (D. stavisagrium) of the shops; the rancorous narcotic qualities of which prohibit its internal use altogether.

Polyandria Polygnia.

10. Zostera. *Z. marina.*

*Ang.* Sea Grasswrack.

*Gen. Desc.* Spike-stalk, strap-shaped, concealed within a grass-like leaf, bearing the fructifications on one side. Cal. 0. Bloss. 0. Stam. alternate. Caps. alternate, seeds solitary.

*Spec. Desc.* *Seed vessel* sitting. *Stem.* much branched. *Leaves* floating, long grass-like, blunt; leaf-scales sheathing pointed. *Flowers* in a cavity three or four inches long, on one side of the leaf near the base. *Sea shores.* Bl. June, Aug.

*Use* This plant, which is thrown on the sea shores in great abundance by the tide, is made use of to build mounds or walls for the purpose of opposing the encroachments of the sea. Exposure to the weather bleaches it white.

white. Buildings are thatched with the green leaves, and it will endure upwards of a century. It is used by the inhabitants of Gothland, in Sweden, as a manure; and is also employed by them for stuffing beds, in preference to hay, as being softer. Horses and swine eat it; cows are not fond of it.—*Withering.*

11. Arum. *A. maculatum. A. minus. A. vulgare.*

*Ang.* Wakerobin. Cuckowpin. Lords and Ladies. Arum.

*Gen. Desc.* Sheath one-leaf, cone-shaped. Fruit-stalk naked above, bearing germens at its bottom, and stamens in the middle.

*Spec. Desc.* *Leaves* halberd-shaped, v. entire, spotted with black, sometimes streaked with white. *Spike-stalk* club-shaped, upper part purple, or buff, or mottled buff and purple. *Germ.* green-yellow. *Summ.* purple. *Anth.* two open cells. *Nectaries,* the row above the stamens exactly similar to the upper germens. *Sheath* pale green. *Berries* in a naked cluster, red. *Shady places.* Bl. May.

*Use.* The root is the medicinal part of this plant, which in a recent and lactescent state is extremely acrimonious; and, upon being chewed, excites an intolerable sensation of burning and pricking in the tongue, which continues several hours: cut in slices and applied to the skin, it has been known to produce blisters. This acrimony is gradually lost by drying, and may be so far dissipated by the application of heat, as to leave the root a bland farinaceous aliment; its medical efficacy, therefore, residing wholly in its active volatile matter, the powdered root can have very little power, and is now very properly omitted in the Mat. Med. This root is considered by Dr. Cullen as a general stimulant, not only exciting the activity of the digestive powers, where they are languid, but stimulating the whole system; in proof of this he observes, that it has been useful in intermittent fevers. Bergius enumerates its uses in many diseases, but he speaks particularly of its efficacy in *Cephalæa sympatica*; in which, though all other means were ineffectual, the powder of this root never failed to give relief: *M. M.* 722. Arum is certainly a very powerful stimulant, and by promoting the secretions may be advantageously employed in *cachectic* and *chlorotic* cases, in *rheumatic* affections, and in various other complaints of phlegmatic and torpid constitutions; but more especially in a weak and relaxed state of the stomach, owing to viscid mucus. If given in powder, care should be taken the root be young and newly dried; when it it may be used in the dose of a scruple, or more, twice a day; but in rheumatisms, and other disorders requiring its full effects, it should be given in a recent state; and to cover its insupportable pungency on the tongue, in the form of an emulsion, with gum arabic and spermaceti, increasing the dose from ten grains to upwards of a scruple, four times a day. This way it occasions a slight sensation of warmth about the stomach, and promotes perspiration plentifully: it has thus removed obstinate rheumatic pains, and is recommended to further trial.—*Woodville.* The roots are esteemed useful in *asthmatic* complaints and obstructions of the bronchiæ; but it is rarely used in the present practice.—*Lightfoot.* There is no doubt but the acrid quality of this plant may be turned to useful purposes, but its proper dose must be first ascertained: as it loses its acrimony by drying, it is probable that the *pulvis ari* owes its virtue chiefly to other ingredients. The root and leaves, when recent, are so extremely acrid, that it is highly disagreeable to taste them; but this quality is wholly extracted either by boiling or baking, and the roots then afford a very wholesome nutriment. Bergius says, that this, and other species of the arum, are made use of as *bread* in several countries, and contain much farinaceous matter: many nations prepare the only bread they have, from plants full as acrimonious, first dissipating by the force of heat the noxious qualities. Starch may be made from the roots. The root dried and powdered, is used by the French for washing the skin, and is sold at a high price, under the name of *Cypress powder*. It is undoubtedly a good and an innocent cosmetic.—*Withering.* They are also said to possess a *saponaceous* quality, and have been used in washing linen, to supply the place of soap.—*Ray. Hist. p.* 1208. The berries ripen about the close of the summer.—*Curtis.*

12. Anemone. *A. pratensis. Pulsatilla nigricans. Ang.* Dark flowered Anemone.

*Gen. Desc.* Cal. generally 0. Pet. five to ten. Caps. with awns, or tails, formed by the style.

*Spec. Desc. Fruit-stalks* with an involucrum. *Seeds* with tails. *Leaves* doubly winged. *Petals* reflex. *Flower* nodding, smaller and darker than *A. pulsatilla*. *Dry hills.* Bl. April.

N. B. It seems doubtful whether this plant be indigenous.

*Use.* This plant, though in its recent state it has scarcely any smell, is to the taste extremely acrid; and, when chewed, chewed, corrodes the tongue and fauces; dried, it retains a considerable share of acrimony. It appears also, from experiments, to contain a camphoraceous matter, which was obtained in the form of chrystals, of an unctuous taste, and very inflammable. It has been received into the Edinburgh Mat. Med. on the authority of Baron Stoerck, who recommends it as an effectual remedy for most of the chronic diseases of the *eye*, particularly *amaurosis, cataract,* and *opacity of the cornea,* proceeding from various causes. Stoerck likewise found it of great use in *venereal nodes, nocturnal pains, ulcers, caries, indurated glands, suppressed menses, serpiginous eruptions, melancholy,* and *palsy:* he experienced, in his own person, its benefit in a contusion of the eye, from which he had suffered for two years; and many German physicians have since testified its good effects in the diseases of the eyes; though by others its efficacy has been denied. Dr. Cullen, nevertheless, recommends a repetition of trials of it in the amaurosis, a disease often otherwise incurable, and dependant upon many different causes. *See M. M. v. 2, p.* 216. Every part of the plant, except the root, is ordered for medicinal use, and was prepared by S. into an extract, a distilled water, and an infusion; but the first form was preferred, and was given seven grains to twenty or thirty, three or four times a day. In large doses it frequently excited nausea and vomiting, or produced griping pains of the bowels and looseness, and very generally proved diuretic. The fluid preparations are likewise recommended externally in ulcers and complaints of the skin. *Woodville.*

( To be continued. )

